# Validation and update of a multivariable prediction model for the identification and management of patients at risk for hepatocellular carcinoma

**DOI:** 10.1186/s12014-021-09326-w

**Published:** 2021-08-19

**Authors:** Bo Li, Youyun Zhao, Wangxi Cai, Anping Ming, Hanmin Li

**Affiliations:** 1 Clinical Laboratory, Hubei Provincial Hospital of Traditional Chinese Medicine, Affiliated Hospital of Hubei University of Traditional Chinese Medicine, Hubei Province Academy of Traditional Chinese Medicine, Wuhan, China; 2 Institute of Hepatology, Hubei Provincial Hospital of Traditional Chinese Medicine, Affiliated Hospital of Hubei University of Traditional Chinese Medicine, Hubei Province Academy of Traditional Chinese Medicine, Wuhan, China; 3 Theory and Application Research of Liver and Kidney in Traditional Chinese Medicine, Hubei Provincial Key Laboratory, Cell Molecular Biology Laboratory, Level 3 Laboratory of Traditional Chinese Medicine Research, State Administration of Traditional Chinese Medicine, 4 Huayuanshan, Yanzhi Road, Liangdao Street, Wuchang District, Hubei 430061 Wuhan, China

**Keywords:** Hepatocellular carcinoma, Prothrombin induced by vitamin K absence-II, Alpha-fetoprotein, Nomogram, Calibration, Logistic regression model

## Abstract

**Background:**

A hepatocellular carcinoma (HCC) prediction model (ASAP), including age, sex, and the biomarkers alpha-fetoprotein and prothrombin induced by vitamin K absence-II, showed potential clinical value in the early detection of HCC. We validated and updated the model in a real-world cohort and promoted its transferability to daily clinical practice.

**Methods:**

This retrospective cohort analysis included 1012 of the 2479 eligible patients aged 35 years or older undergoing surveillance for HCC. The data were extracted from the electronic medical records. Biomarker values within the test-to-diagnosis interval were used to validate the ASAP model. Due to its unsatisfactory calibration, three logistic regression models were constructed to recalibrate and update the model. Their discrimination, calibration, and clinical utility were compared. The performance statistics of the final updated model at several risk thresholds are presented. The outcomes of 855 non-HCC patients were further assessed during a median of 10.2 months of follow-up. Statistical analyses were performed using packages in R software.

**Results:**

The ASAP model had superior discriminative performance in the validation cohort [C-statistic = 0.982, (95% confidence interval 0.972–0.992)] but significantly overestimated the risk of HCC (intercept − 3.243 and slope 1.192 in the calibration plot), reducing its clinical usefulness. Recalibration-in-the-large, which exhibited performance comparable to that of the refitted model revision, led to the retention of the excellent discrimination and substantial improvements in the calibration and clinical utility, achieving a sensitivity of 100% at the median prediction probability of the absence of HCC (1.3%). The probability threshold of 1.3% and the incidence of HCC in the cohort (15.5%) were used to stratify the patients into low-, medium-, and high-risk groups. The cumulative HCC incidences in the non-HCC patients significantly differed among the risk groups (log-rank test, p-value < 0.001). The 3-month, 6-month and 18-month cumulative incidences in the low-risk group were 0.6%, 0.9% and 0.9%, respectively.

**Conclusions:**

The ASAP model is an accurate tool for HCC risk estimation that requires recalibration before use in a new region because calibration varies with clinical environments. Additionally, rational risk stratification and risk-based management decision-making, e.g., 3-month follow-up recommendations for targeted individuals, helped improve HCC surveillance, which warrants assessment in larger cohorts.

**Supplementary Information:**

The online version contains supplementary material available at 10.1186/s12014-021-09326-w.

## Background

Liver cancer is the seventh most common malignant tumor and the second leading cause of death among all cancers worldwide [1]. It is one of the five leading causes of death in the Chinese population, among which hepatocellular carcinoma (HCC) accounts for more than 85–90%. The development of HCC is closely related to chronic liver injury of any etiology. Early HCC detection is of paramount importance for improving prognosis due to the lack of a specific clinical presentation [2]. Combined testing of tumor markers can increase the sensitivity without reducing the specificity of the diagnosis [3]. Scientific risk-based stratification is a key means of improving the overall survival rate [4].

Increasing numbers of models have been derived to predict the risk of HCC, which can then be used to stratify patients. Some models require unique indicators (e.g., genetic testing or DNA level) that make data collection difficult or are limited to a certain group of people, such as those with viral infections, those taking antivirals, and those with a specific alpha-fetoprotein (AFP) level, substantially limiting the external verification and universality of such models [5–7]. An easy-to-use, international prediction model is urgently needed to guide the personalized management of populations at risk for HCC.

Recently, a simple multicenter collaborative and verified model for the prediction of HCC in hepatitis B virus (HBV)-infected patients was reported and had promising discrimination ability. The ASAP model includes four factors: age, sex, AFP level, and prothrombin induced by vitamin K absence-II (PIVKA-II) level. The online calculator can predict the probability of target patients developing HCC and classify them into three risk groups. The accompanying surveillance decisions include the recommendation that low-risk individuals do not need to undergo imaging at 6-month intervals [8]. Moreover, a retrospective case-control study reported that the best model containing the same four factors improved the accuracy of the detection of early-stage HCC in patients with viral or nonviral chronic liver disease predominantly in populations of Caucasian and African American descent [9]. However, this model has not been validated in real-world clinical practice. When prediction models are validated in new circumstances, due to differences in the case mix and model predictor effects, miscalibration is common, leading to reduced utility [10, 11]. Therefore, comprehensive model validation, including an assessment of the discrimination ability, calibration, and clinical application, is indispensable.

This study was performed to validate and update the ASAP model in patients with viral or nonviral chronic hepatitis and cirrhosis in daily clinical practice and explore the appropriate thresholds for the stratification of risk groups that can be used to inform the selection of health management strategies.

## Methods

### Data collection

This retrospective study was performed in an academic tertiary hospital, Hubei Provincial Hospital of Traditional Chinese Medicine in Central China, from May 2018 to January 2021. Data were extracted from the electronic medical records. Records from consecutive inpatients with both AFP and PIVKA-II results were reviewed. Data from patients older than 35 years with hepatitis, cirrhosis, hepatic benign space-occupying lesions (SOLs), or HCC before October 2019 were included.

The levels of AFP and PIVKA-II were measured with the appropriate testing systems from Roche Diagnostics (Shanghai, China) and Fujirebio Diagnostics (Fujirebio, Japan), respectively. Because the levels of AFP and PIVKA-II can rapidly increase with the onset of HCC, we defined three weeks as the longest time from the marker measurements to HCC diagnosis [12–14] and selected the first detected values to validate and update the ASAP model. Individuals with undiagnosed HCC beyond that interval and those without HCC were considered at-risk individuals in the subsequent follow-up analysis. The outcome was the definitive diagnosis of HCC. The follow-up duration was calculated from the time of the first measurement to the date of the conclusive HCC diagnosis or January 31, 2021. Data were censored at the time of loss to follow-up, the time of non-HCC-related death, and the end of the study.

The diagnosis of HCC was refuted or confirmed based on a comprehensive reference standard, and both a clinical diagnosis and pathological diagnosis were made based on spiral computed tomography, magnetic resonance imaging, and liver biopsy [4].

### Missing data

There were no missing data for AFP or PIVKA-II.

### Statistical analysis

The levels of AFP and PIVKA-II were log transformed because they had right-skewed distributions.

Based on the published coefficient values in the development cohort [8], we calculated the linear predictor of each patient in the validation cohort as an offset variable with the coefficient set to 1 and intercept set to 0 to validate the ASAP model. The linear predictor was calculated as follows: − 7.57711770 + 0.04666357[age] − 0.57611693[sex] + 0.42243533[log(AFP)] + 1.10518910[log(PIVKA-II)] [8]. We evaluated its discrimination, calibration, and clinical benefit and then recalibrated and updated it based on three logistic regression models [15]. Recalibration-in-the-large included the ASAP linear predictor with coefficient set to 1 and the assessed intercept. In recalibration, we evaluated the intercept and the coefficient of the ASAP predictor. In model revision, we kept all variables in the original ASAP model and refitted it. We used a bootstrap resampling procedure in the complete updated samples to correct for optimism when model revision was applied. The overall performance was evaluated by the Brier score and compared among models by the chi-square test. A lower Brier score indicates better performance or closer to being correct. The final updated model was selected according to a previously reported procedure as follows. If the test of the model revision against the original ASAP model was not significant, we adopted the original ASAP model; otherwise, we continued. If the test of the model revision against recalibration-in-the-large was not significant, we adopted the updated model intercept; otherwise, we continued. If the test of the model revision against recalibration was not significant, we adopted the recalibration model; otherwise, we adopted the revised model [15].

Discrimination was measured using the C-statistic. Calibration was assessed with calibration plots. The classification accuracy performances were measured using the sensitivity, specificity, positive predictive value (PPV), and negative predictive value (NPV) at a selection of thresholds. The net benefit (NB) of the model was evaluated with decision curve analysis.

We chose the median prediction value of the non-HCC cases in the final updated model and the HCC event rate in the validation cohort from a list of common thresholds to classify the patients into low-, medium-, and high-risk groups [10].

The differences in the relative risk ratios between two risk groups were compared by the chi-square test. The cumulative HCC incidences of the different risk groups without HCC were obtained by the Kaplan-Meier method, and their discrepancies were compared by the log-rank test.

The model established in our study complied with the Transparent Reporting of a multivariable prediction model for Individual Prognosis Or Diagnosis (TRIPOD) guidelines [12] (see Additional file 1: Table S1).

A p-value less than 0.05 was considered statistically significant. We carried out statistical analyses using packages in R software (v3.6.2 and v4.0.2; R Foundation for Statistical Computing).

## Results

### Participant selection and characteristics

**Fig. 1:**
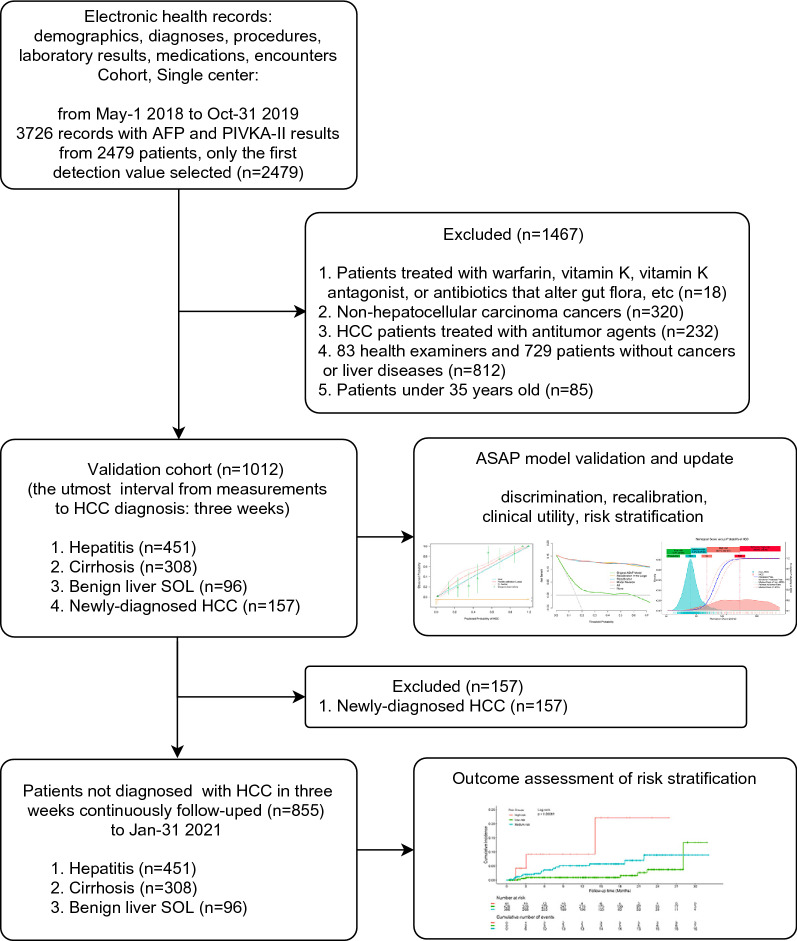
Analysis flowchart. Abbreviations: AFP, alpha-fetoprotein; HCC, hepatocellular carcinoma; PIVKA-II, prothrombin induced by vitamin K absence-II; SOL, space-occupying lesion

In total, 3726 records of 2479 participants were reviewed; 1247 records were repeated measurements and, thus excluded. In total, 2479 patients with the first detection values of biomarkers were further selected. Furthermore, 1467 individuals were excluded for the following reasons: 18 patients treated with warfarin, vitamin K, vitamin K antagonist, or antibiotics that alter the gut flora; 320 non-HCC cancers; 232 HCC patients treated with antitumor agents; 83 health examiners and 729 patients without cancers or liver diseases; and 85 patients with hepatitis aged under 35 years. Ultimately, 1012 qualified patients were included in the model validation process. After excluding 157 confirmed HCC cases, 855 at-risk patients were included in the continued follow-up analysis to compare the outcomes across the different risk groups (Fig. 1). The validation cohort was dominated by single HBV-infected patients (77.3%), who had an HCC incidence rate of 15.6% (122/782), while the incidence in patients with other etiologies was 15.2% (35/230). There was no significant difference between the two incidences (χ2 p-value = 0.967). The overall HCC incidence in the validation cohort was 15.5%, which was significantly lower than that in the development cohort (41.1%) reported by Yang et al while deriving the original ASAP model [8]. Table 1 shows the basic characteristics of the two cohorts. Due to the different patient selection criteria and data collection methods from respective distinct clinical settings, there were significant differences in age, etiology, case composition, Child-Pugh class, bilirubin, alanine aminotransferase, and albumin between the development and validation cohorts. The AFP and PIVKA-II serum levels are shown in Additional file 2: Fig. S1a, b.

**Table 1:** Baseline characteristics of the study participants compared with the original development cohort

Parameters	Development cohort^1^					Validation cohort				
	Hepatitis	Cirrhosis	Benign SOL	HC	HCC	Hepatitis		Cirrhosis	Benign SOL	HCC
	(n = 289)	(n = 314)	(n = 179)	(n = 508)	(n = 908)	(n = 451)		(n = 308)	(n = 96)	(n = 157)
Age, mean (SD), years	50.2 (13.3)	45.1 (13.6)	52.2 (13.1)	41.2 (11.9)	53.5 (10.1)	50.7 (9.4)		58.1 (10.7)	58.5 (12.4)	59.6 (120)
Sex, n (%)										
Male	195 (67.5%)	213 (67.8%)	101 (56.4%)	252 (49.6%)	752 (82.8%)	345 (76.5%)		206 (66.9%)	58 (60.4%)	133 (84.7%)
Female	94 (32.5%)	101 (32.2%)	78 (43.6%)	256 (50.4%)	156 (17.2%)	106 (23.5%)		102 (33.1%)	38 (39.6%)	24 (15.3%)
Etiology, n (%)										
HBV	289 (100%)	289 (100%)	/	0 (0%)	289 (100%)	430 (95.3)		194 (63.0)	36 (37.5)	122 (77.7)
HCV	0 (0%)	0 (0%)	/	0 (0%)	0 (0%)	4 (0.9)		27 (8.8)	6 (6.3)	10 (6.4)
Alcoholic	0 (0%)	0 (0%)	/	0 (0%)	0 (0%)	3 (0.7)		33 (10.7)	5 (5.2)	7 (4.4)
Others (Schistosomiasis, HBV & HCV overlapping infection, HEV)	0 (0%)	0 (0%)	/	0 (0%)	0 (0%)	14 (3.1)		54 (17.5)	49 (51.0)	18 (11.5)
BCLC stage, 0+A/B+C/D, n (%)					318 (35.0)/590 (65.0)/0 (0)					32 (20.4)/99 (63.0)/26 (16.6)
Child-Pugh class, n (%)										
A	289 (100)	312 (99.4)	175 (97.8%)	508 (100%)	903 (99.4)	414 (91.8)		192 (62.3)	71 (74)	68 (43.3)
B	0	2 (0.6)	4 (2.2%)	0	4 (0.4)	29 (6.4)		95 (30.9)	23 (24)	53 (33.8)
C	0	0	0	0	1 (0.2)	8 (1.8)		21 (6.8)	2 (2.0)	36 (22.9)
Total bilirubin, mean (SD), μmol/L	15.1 (12.2)	56.6 (88.4)	18.0 (21.3)	16.2 (9.2)	16.9 (36.1)	27.6 (75.6)		47.7 (43.2)	32.3 (59.7)	79.9 (133.6)
Platelet, mean (SD), 10^9^/L	193.7 (69.8)	117.4 (71.4)	226.6 (104.3)	189.2 (46.5)	169.8 (77.7)	168.1 (59.8)		105.6 (70.0)	150.5 (72.3)	147.6 (85.4)
Alanine aminotransferase, mean (SD), U/L	35.8 (45.7)	122.9 (268.1)	42.2 (64.5)	34.7 (34.6)	43.4 (56.4)	80.1 (359.9)		47.4 (126.7)	73.0 (177.8)	89.8 (273.6)
Aspartate aminotransferase, mean (SD), U/L	/	/	/	/	/	60.3 (197.5)		53.6 (88.3)	64.9 (145.2)	134.3 (178.0)
Gamma-glutamyltransferase, mean (SD), U/L	/	/	/	/	/	48.6 (90.0)		77.9 (122.5)	78.1 (110.9)	211.2 (217.7)
Gamma-glutamyltransferase/aspartate aminotransferase ratio, mean (SD)	/	/	/	/	/	1.31 (1.91)		1.70 (2.05)	1.64 (1.52)	2.35 (2.49)
Prothrombin time, mean (SD), s	11.5 (0.9)	14.5 (3.5)	11.4 (1.4)	11.5 (0.7)	11.9 (1.1)	13.7 (7.7)		14.6 (4.8)	12.8 (3.0)	14.5 (5.5)
International normalized ratio, mean (SD)	0.96 (0.07)	1.24 (0.31)	0.95 (0.09)	0.97 (0.04)	1.03 (0.45)	1.26 (0.68)		1.34 (0.44)	1.19 (0.28)	1.34 (0.49)
Albumin, mean (SD), g/L	42.0 (3.7)	38.5 (5.7)	42.6 (4.1)	42.8 (4.7)	42.2 (4.7)	43.0 (5.4)		35.5 (7.2)	38.2 (6.1)	32.6 (6.4)
Diabetes mellitus, n (%)	4 (1.4)	8 (2.5)	8 (4.5)	0 (0)	71 (7.8)	1 (0.2)		21 (6.8)	2 (2.1)	17 (10.8)
AFP, mean (SD), log ng/mL	/	/	/	/	/	1.46 (1.09)		1.70 (1.32)	1.87 (1.55)	5.94 (3.85)
PIVKA-II, mean (SD), log mAU/mL	/	/	/	/	/	3.14 (0.58)		3.33 (1.00)	3.54 (1.08)	8.47 (2.56)
HBeAg-negative, (%, n)	/	/	/	/	/	(74.6, 406)		(86.6, 186)	(82.9, 35)	(84.2, 114)
HBV DNA, < 50 IU/ml, (%, n)	/	/	/	/	/	(56.8, 382)		(74.5, 188)	(56.3, 32)	(20.2, 119)

*AFP* alpha-fetoprotein, *BCLC* Barcelona Clinic Liver Cancer, *SOL* space-occupying lesion, *HBeAg* hepatitis B virus e antigen, *HBV* hepatitis B virus, *HC* healthy control, *HCC* hepatocellular cancer, *HCV* hepatitis C virus, *HEV* hepatitis E virus, *PIVKA*-*II* protein induced by vitamin K absence or antagonist-II, *SD* standard deviation

^1^The data were summarized from the original report of the derivation of the ASAP model reported by Yang et al [8]; "/" indicates not applicable or no data available

### External model validation

Table 2 shows the characteristics and performance of the models considered in this paper. The models contained four predictor factors: age (years, in 1-year increments), sex (male = 0, female = 1), AFP (log ng/mL), and PIVKA-II (log mAU/mL). b_age_, b_sex_, b_AFP_, and b_PIVKA-II_ were the regression coefficients that indicated how a patient’s values of the predictor factors affected the risk of HCC. Regarding b_age_, which was 0.04666357 in the original ASAP model [8], the odds ratio of age was exp(b_age_), i.e., 1.048. The increased risk of HCC among the patients was 0.048 per year after adjusting for other factors. Regarding the recalibration-in-the-large, including the ASAP linear predictor with the coefficient set to 1, b_age_ was the same as that of the ASAP model. Regarding the recalibration assessed with a slope of 1.192, b_age_ was 0.0556229754, equal to 0.04666357 multiplied by 1.192. In the model revision, we refitted it with our data, and the estimate of b_age_ was 0.06178. Therefore, different models generated different b_age_ values. The same was true for the diverse regression coefficients of other factors in different models. The negative b_sex_ illustrated that the female patients had a lower risk of HCC than the reference group male patients after adjusting for age, AFP, and PIVKA-II. In the recalibration-in-the-large, the b_sex_ was − 0.57611693. The odds ratio of sex was 0.562. Female patients had a 43.8% lower risk of HCC than male patients after adjusting for other variables in the model.

**Table. 2:** Characteristics and performance of the original and updated ASAP diagnostic prediction models

Characteristics	ASAP model^1^	Recalibration-in-the-large	Recalibration	Model revision
Calibration parameters				
Intercept	0	− 3.243	− 3.577	–
Slope	1	1	1.192	–
Model coefficients^2^				
Intercept	− 7.57711770	− 10.82011770	− 12.60892	− 12.95521
b_age_	0.04666357	0.04666357	0.0556229754	0.06178
b_sex_	− 0.57611693	− 0.57611693	− 0.6867313806	− 0.7449
b_AFP_	0.42243533	0.42243533	0.5035429134	0.55643
b_PIVKA-II_	1.10518910	1.10518910	1.3173854072	1.28268
Model performance				
Calibration intercept	− 3.243	− 0.000	− 0.000	− 0.000
Calibration slope	1.192	1.192	1	1
Residual deviance	851.25	212.552	208.489	207.962
df	1012	1011	1010	1007
LRT chi-square p-value^3^	< 0.001	0.332	0.913	–
Emax (95% CI)	0.526 (0.483–0.557)	0.053 (0.042–0.159)	0.087 (0.049–0.198)	0.082 (0.043–0.185)
Eavg (95% CI)	0.246 (0.230–0.262)	0.009 (0.007–0.018)	0.006 (0.004–0.015)	0.005 (0.004–0.015)
C-statistic (95% CI)	0.982 (0.970–0.990)	0.982 (0.972–0.991)	0.982 (0.971–0.991)	0.982 (0.971–0.991)
Brier score	0.127107476	0.028182937	0.0280551	0.02789345
AIC	851.25	214.55	212.49	217.96

*AFP* alpha-fetoprotein, *AIC* akaike information criterion, *CI* confidence interval, *df* degrees of freedom, *Eavg* average absolute difference in the predicted and calibrated probabilities, *Emax* maximum absolute difference in the predicted and calibrated probabilities, *LRT* likelihood ratio test, *PIVKA*-*II* protein induced by vitamin K absence or antagonist-II

^1^The model coefficients are derived from the original ASAP model reported by Yang et al [8]

^2^b_age_, b_sex_, b_AFP_, and b_PIVKA-II_ are the regression coefficients of age, sex, AFP, and PIVKA-II, respectively

^3^Comparison with the model revision using the methods described by Vergouwe et al [15]

**Fig. 2:**
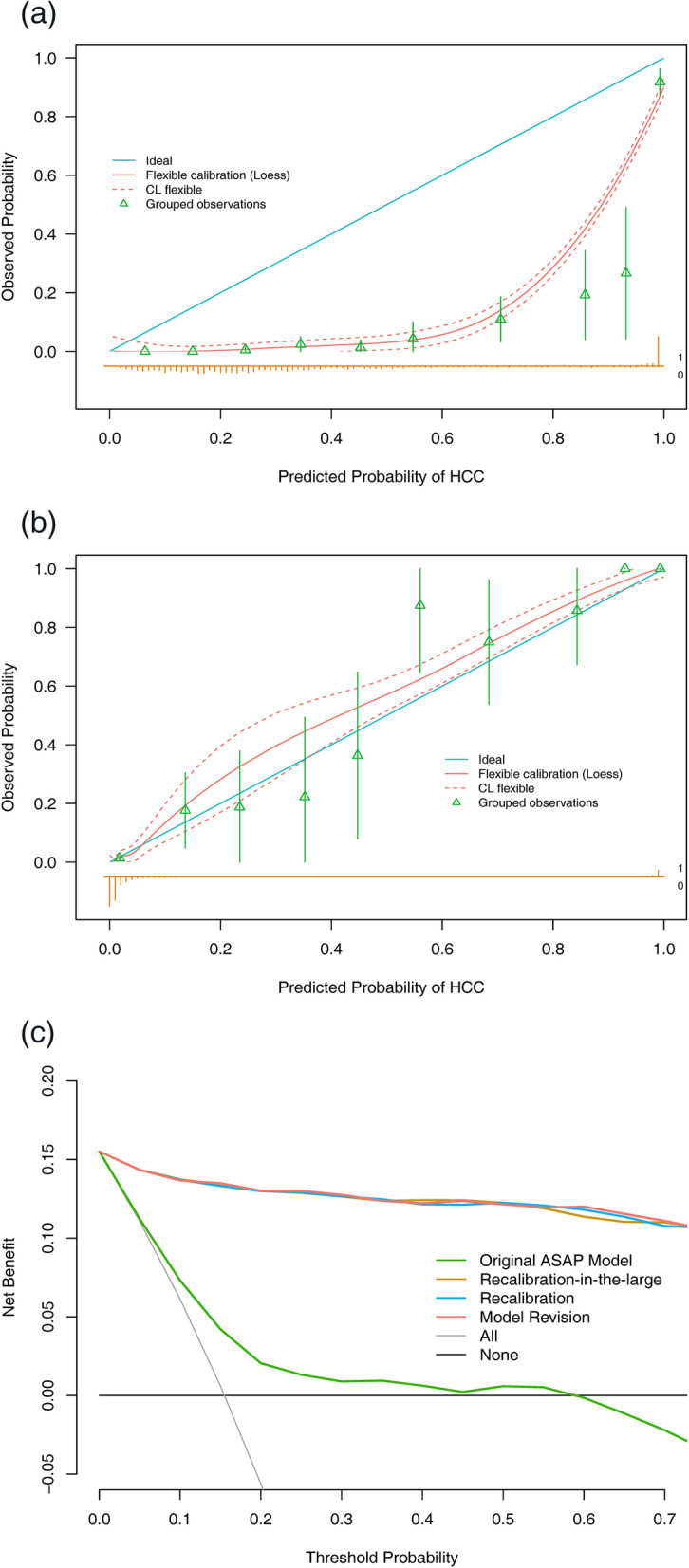
Calibration and decision curves of the ASAP model and the updated models predicting HCC risk. Calibration curves of the predicted probabilities versus the observed probabilities of (**a**) the original ASAP model reported by Yang et al [8]; and (**b**) recalibration-in-the-large developed based on the method described by Vergouwe et al [15] in the validation cohort (n = 1012). A nonparametric calibration curve with the 95% confidence limits (CL) (red slide line with dashed lines) was created with the Loess algorithm. Observed HCC occurrence (green triangles) with the 95% CL was plotted against the average predicted probability in each group. The blue straight diagonal line serves as a reference for perfect calibration. The brown bar chart at the bottom of the figure presents the distribution of the predicted probabilities of the cases with outcomes (above the line) and those without outcomes (below the line) (“1” vs. “0”). (**c**) Decision curves showing the net benefit correlated with the utility of the original ASAP model reported by Yang et al [8] and the updated models (recalibration-in-the-large, recalibration, and model revision) derived using the methods described by Vergouwe et al [15]. Notably, the coefficients of the models are listed in Table 2. The logit (P) calculation formula of the ASAP model was {− 7.57711770 + 0.04666357[age] - 0.57611693[sex]+ 0.42243533[log(AFP)] + 1.10518910[log(PIVKA-II)]}; the formula of recalibration-in-the-large was {-10.82011770 + 0.04666357[age] − 0.57611693[sex] + 0.42243533[log(AFP)] + 1.10518910[log(PIVKA-II)]}; the formula of recalibration was {− 12.60892 + 0.0556229754[age] − 0.6867313806[sex] + 0.5035429134[log(AFP)] + 1.3173854072[log(PIVKA-II)]}; and the formula of the model revision was {-12.95521 + 0.06178[age] − 0.7449[sex]+ 0.55643[log(AFP)] + 1.28268[log(PIVKA-II)]}. Abbreviations: AFP, alpha-fetoprotein; CL, confidence limits; HCC, hepatocellular carcinoma; Loess: locally weighted linear regression; PIVKA-II, prothrombin induced by vitamin K absence-II

The ASAP model had a high diagnostic accuracy {C-statistic = 0.982, [95% confidence interval (CI), 0.972–0.992]} but significantly overestimated the risk of HCC (intercept − 3.243 and slope 1.192 in the calibration plot) (Table 2, Fig. 2a) and had poor clinical utility at the probability thresholds of 1/3 and 2/3 set in the model [8] [with respective NBs of 0.011 (95% CI − 0.017 to 0.038) and − 0.019 (95% CI, − 0.061 to 0.025) in the decision curve analysis] (Fig. 2c).

Recalibration was necessary due to the apparent overfitting of the ASAP model with the new data (Table 2, Fig. 2a). The three updated models had the same excellent discrimination ability (C-statistic = 0.982), and their calibration was superior to that of the ASAP model, as they had nearly zero intercepts. The comparison of the model revision against the ASAP model was significant [likelihood ratio test (LRT) χ2 p-value < 0.001]. Recalibration-in-the-large revealed an intercept of − 3.243 and resulted in a significant improvement in the model fit compared with that of the ASAP model [LRT χ2 p-value = 0.04384 (data not shown)], with a recalibration slope of 1.192, and did not significantly differ from the revised model (LRT χ2 p-value = 0.332). A nonparametric calibration plot using the locally weighted linear regression (Loess) function is shown in Fig. 2b. Recalibration revealed an intercept of − 3.577 and a slope of 1.192. Recalibration further improved the Brier score and average absolute difference in the predicted and calibrated probabilities (Eavg) [16] and did not significantly differ from the revised model (LRT χ2 p-value = 0.913), with a recalibration slope close to 1. Calibration curves of the recalibration and model revision are shown in Additional file 3: Fig. S2a, b. The model revision improved the calibration the most, with the smallest Brier score and Eavg (Table 2). The bootstrap resampling procedure was used to validate the revised model as there was some overfitting with an optimism-corrected C-statistic of 0.981 and a shrinkage factor of 0.976. The nomogram and score tables of the re-estimated ASAP model are shown in Additional file 4: Fig. S3a and Additional file 5: Table S2, respectively.

Based on the closed testing procedure [15], the ASAP model significantly differed from the model revision (LRT χ2 p-value < 0.001), but recalibration-in-the-large showed no significant difference (LRT χ2 p-value = 0.332); recalibration-in-the-large was adopted for the final updated model by adding an intercept of − 3.243 to the ASAP linear predictor (Table 2).

In addition, the decisive curve of recalibration-in-the-large nearly completely overlapped with those of recalibration and model revision, with high NBs over the original model within the entire threshold range (Fig. 2c). At the 1/3 risk threshold, it increased the NB by 0.115 over the ASAP model, representing 11.5 more HCC cases identified per 100 patients for the same number of unnecessary interventions (Table 3) [17]. It is relatively more beneficial for clinicians to use the updated model to assess at-risk individuals and improve clinical decision-making.

**Table. 3:** Classification statistics of the selected thresholds for the recalibration-in-the-large of the validation cohort and subsets

Probability threshold^1^	Proportions divided		Sensitivity (95% CI)^2^	Specificity (95% CI)^2^	PPV (95% CI)^2^	NPV (95% CI)^2^	NB (95% CI)^2^
	All patients	HCC patients					
For HCC of any etiology, [15.5% (157/1012)]							
1.3%	427/685	0/157	1 (1–1)	0.499 (0.466–0.532)	0.268 (0.256–0.282)	1 (1–1)	0.150 (0.125–0.173)
13.1%	823/189	13/144	0.917 (0.873–0.955)	0.947 (0.931–0.963)	0.763 (0.71–0.818)	0.984 (0.976–0.992)	0.136 (0.113–0.160)
15.5%	829/183	15/142	0.905 (0.854–0.949)	0.952 (0.937–0.966)	0.777 (0.724–0.832)	0.982 (0.973–0.99)	0.133 (0.112–0.155)
1/3	856/156	20/137	0.873 (0.815–0.924)	0.978 (0.966–0.987)	0.879 (0.828–0.927)	0.977 (0.967–0.986)	0.126 (0.104–0.145)
50%	874/138	26/131	0.834 (0.771–0.892)	0.992 (0.985–0.998)	0.950 (0.909–0.985)	0.970 (0.960–0.980)	0.123 (0.102–0.143)
2/3	888/124	37/120	0.764 (0.694–0.828)	0.995 (0.991–0.999)	0.968 (0.934–0.992)	0.958 (0.947–0.969)	0.111 (0.090–0.132)
98.3%	933/79	78/79	0.503 (0.427–0.580)	1 (1–1)	1 (1–1)	0.916 (0.905–0.928)	0.078 (0.061–0.095)
For single HBV-related HCC, [15.6% (122/782)]							
1.3%	367/415	0/122	1 (1–1)	0.556 (0.520–0.596)	0.294 (0.278–0.314)	1 (1–1)	0.151 (0.129–0.178)
13.1%	646/136	8/114	0.934 (0.885–0.975)	0.967 (0.952–0.979)	0.839 (0.782–0.892)	0.988 (0.979–0.995)	0.142 (0.120–0.168)
15.5%	652/130	10/112	0.918 (0.869–0.959)	0.973 (0.959–0.985)	0.862 (0.807–0.916)	0.985 (0.976–0.992)	0.139 (0.114–0.163)
1/3	664/118	13/109	0.893 (0.836–0.943)	0.986 (0.977–0.994)	0.924 (0.875–0.966)	0.980 (0.970–0.989)	0.134 (0.109–0.160)
50%	675/107	19/103	0.844 (0.779–0.910)	0.994 (0.988–0.996)	0.963 (0.924–0.991)	0.972 (0.960–0.984)	0.127 (0.102–0.153)
2/3	685/97	28/94	0.771 (0.697–0.844)	0.995 (0.989–1)	0.969 (0.931–1)	0.959 (0.947–0.972)	0.113 (0.087–0.141)
98.3%	716/66	56/66	0.541 (0.451–0.631)	1 (1–1)	1 (1–1)	0.922 (0.908–0.936)	0.084 (0.064–0.105)
For HCC of other causes, [15.2% (35/230)]							
1.3%	60/170	0/35	1 (1–1)	0.308 (0.246–0.369)	0.206 (0.192–0.222)	1 (1–1)	0.144 (0.100–0.193)
13.1%	177/53	5/30	0.857 (0.743–0.971)	0.882 (0.836–0.928)	0.569 (0.475–0.680)	0.972 (0.949–0.994)	0.115 (0.073–0.159)
15.5%	177/53	5/30	0.857 (0.743–0.971)	0.882 (0.836–0.928)	0.569 (0.475–0.680)	0.972 (0.949–0.994)	0.112 (0.067–0.157)
1/3	192/38	7/28	0.800 (0.657–0.914)	0.949 (0.918–0.974)	0.737 (0.620–0.862)	0.964 (0.939–0.984)	0.100 (0.057–0.148)
50%	199/31	7/28	0.800 (0.657–0.914)	0.985 (0.964–1)	0.906 (0.800–1)	0.965 (0.941–0.985)	0.109 (0.061–0.157)
2/3	203/27	9/26	0.743 (0.600–0.886)	0.995 (0.985–1)	0.964 (0.880–1)	0.956 (0.932–0.980)	0.104 (0.061–0.152)
98.3%	217/13	22/13	0.371 (0.229–0.543)	1 (1–1)	1 (1–1)	0.899 (0.878–0.924)	0.057 (0.030–0.091)

*CI* confidence interval, *HCC* hepatocellular cancer, *NB* net benefit, *NPV* negative prediction value, *PPV* positive prediction value

^1^1.3%, the median prediction probability of non-HCC; 13.1%, the optimal cutoff value; 15.5%, the incidence rate of HCC in the validation cohort; 1/3, the low threshold in the original ASAP model; 50%, the midpoint of the sigmoid curve; 2/3, the high threshold in the original ASAP model; 98.3%, the median prediction probability of HCC

^2^Computed with 2000 stratified bootstrap replicates

### Performance of recalibration-in-the-large among different etiologies

Table 3 lists the proportions of overall and HCC patients divided by the selected thresholds of recalibration-in-the-large and the statistical characteristics. The thresholds included the optimal cutoff value (13.1%), the incidence in the validation cohort (15.5%), the midpoint of the sigmoid curve (50%), the tierce-points of the sigmoid curve (1/3, 2/3) set in the original ASAP model [8], the median prediction probability in the non-HCC cases (1.3%), and the median prediction probability in the HCC cases (98.3%). All cutoff values were commonly adopted in the model performance evaluation. Lower thresholds resulted in higher sensitivities, lower specificities and higher NBs in the recalibration-in-the-large, and vice versa.

Recalibration-in-the-large functioned slightly better in patients with single HBV infections, with a C-statistic of 0.990 (95% CI 0.984–0.997), than in individuals with other etiologies, with a C-statistic of 0.943 (95% CI 0.894–0.992) (Z = 1.879, p-value = 0.060). From probability thresholds of 0 to 0.7, the single HBV-infected group presented consistently higher NBs than the remaining group (Table 3), equivalent to detecting more HCC cases without increasing the number of false positives [17].

### Risk threshold selection and risk stratification

Risk stratification is the cornerstone of the individualized management of patients at risk for HCC. However, an optimal risk threshold does not exist for a prediction model. Reasonable risk thresholds should be selected based on the actual clinical setting. Considering the severity of the disease, low thresholds that are less than 50% and have high NPVs are usually preferred [10, 18]. Similar to the distribution of the predicted probabilities of HCC and non-HCC patients in the model revision (Additional file 4: Fig. S3b), the recalibration-in-the-large achieved a sensitivity of 100% and an NPV of 100% at a probability threshold of 1.3% and a specificity of 100% and a PPV of 100% at 98.3% with regard to the diagnosis of HCC regardless of etiology in the validation cohort (Table 3). From the listed thresholds, we selected probability thresholds of 1.3% and 15.5% to stratify the validation cohort into low-, medium-, and high-risk groups. As a result, 427 non-HCC patients were classified into the low-risk group; 183 patients, including 142 HCC cases, were classified into the high-risk group; and the other 402 patients, including 15 HCC cases, were sorted into the medium-risk group. The incidence rates in the three groups were 0% (0/427), 3.7% (15/402), and 77.6% (142/183). Taking the medium-risk group as the reference, the relative risk (RR) ratios and 95% CIs of incident HCC were 0.0 in the low-risk group and 20.8 (11.9–36.4) in the high-risk group.

### Clinical outcome assessment after risk stratification

**Fig. 3:**
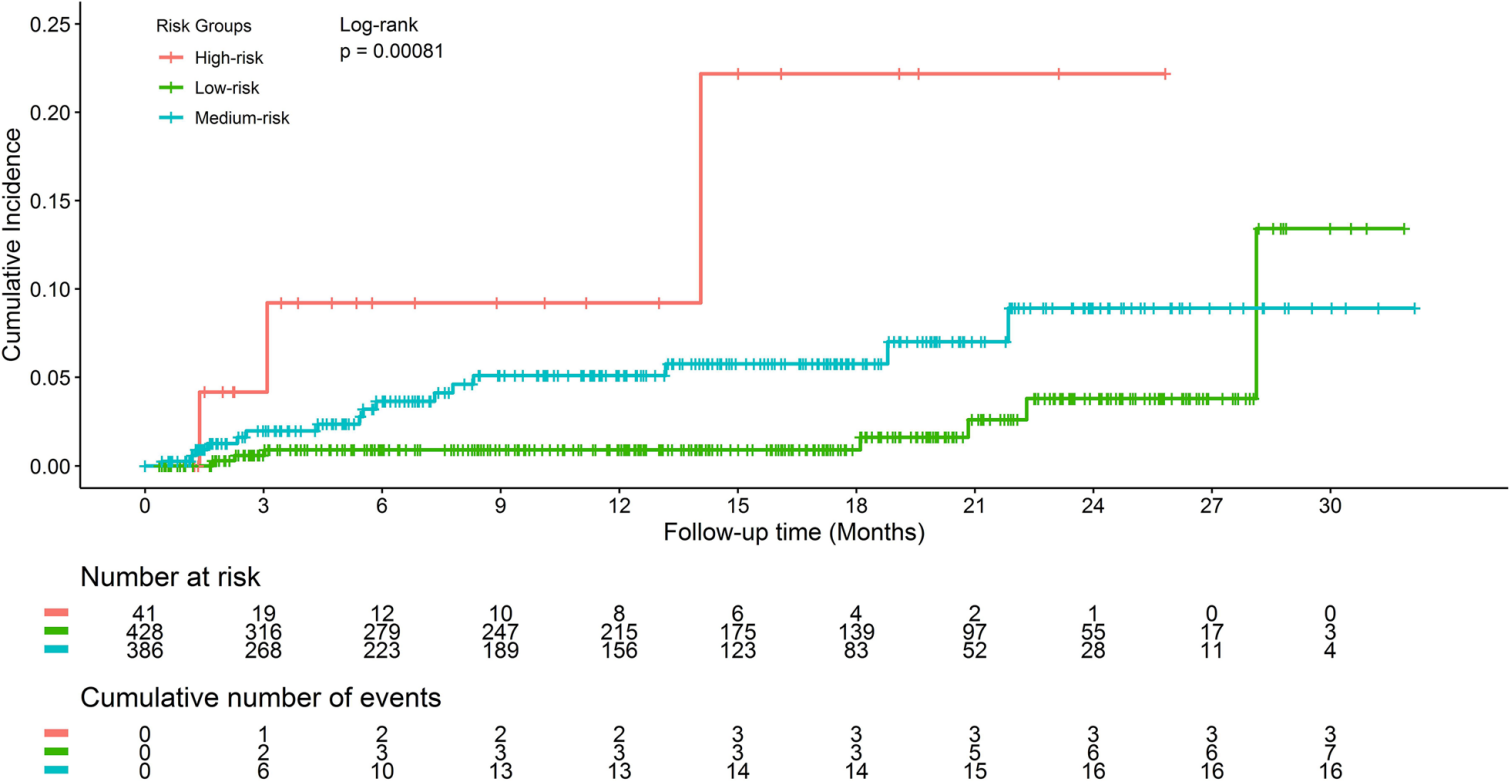
Cumulative HCC incidences in three risk groups based on the recalibration-in-the-large. Kaplan-Meier curve demonstrating significant differences in the cumulative HCC incidences among the low-risk group (predicted probability < 1.3%, the median of the predicted probability in the non-HCC patients), medium-risk group (predicted probability 1.3–15.5%), and high-risk group (predicted probability > 15.5%, the HCC incidence in the validation cohort) (log-rank test, p-value = 9e−04; low-risk vs. medium-risk with p-value = 0.01, medium-risk vs. high-risk with p-value = 0.08, and low-risk vs. high-risk with p-value = 3e−05). The two probability thresholds were reasonable for risk classification based on the estimation by recalibration-in-the-large in the validation cohort. Abbreviations: HCC, hepatocellular carcinoma

After excluding HCC cases with a confirmative diagnosis, the remaining 855 at-risk individuals were continuously observed for incident HCC during a median of 10.2 months of follow-up. The cumulative HCC incidences were significantly different in the low-, medium-, and high-risk groups (log-rank test, p-value < 0.001) (Fig. 3). The low-risk group maintained the lowest incidence over a period of up to two years. The 3-month, 6-month and 18-month cumulative incidences were 0.6%, 0.9% and 0.9% in the low-risk group; 2.0%, 3.6% and 5.8% in the medium-risk group; and 4.2%, 9.2% and 22.2% in the high-risk group.

These results showed that recalibration-in-the-large, combined with reasonable thresholds of 1.3% and 15.5%, could be used to effectively stratify patients based on their need for HCC surveillance into three risk groups that are useful in everyday clinical practice. Patients in the high-risk group are likely to be diagnosed with HCC soon and need fairly intensive surveillance. Patients with uncertain liver SOL in any risk group should undergo close monitoring at 3-month intervals. Patients with definitely benign or absent liver SOL in the low-risk group could undergo monitoring every year, and similar patients in the medium-risk group should return for surveillance at 6-month or shorter intervals.

## Discussion

This study validated the ASAP model [8] in a significantly different setting from that in which it was developed. In this setting, the patients underwent all-cause surveillance for HCC (Table 1). The model was highly miscalibrated, and its uncertainties were corrected using three methods. By comparing the overall performance of these models, the recalibration-in-the-large model was used for the prediction of incident HCC, and two reasonable thresholds were set for the classification management of at-risk patients.

Although the original model was significantly more discriminative in the validation cohort than in the development cohort [C-statistic, 0.982 (95% CI 0.972–0.992) vs. 0.941 (95% CI 0.929–0.952)] [8], it systematically overestimated the probability of HCC (Fig. 2a), resulting in a reduced clinical benefit and possibly even harm (Fig. 2c), including more radiation exposure, more psychological harm, and higher costs [10, 11, 19–21]. The main reason may be the difference in case mix in the two cohorts [11]; the average increased prediction probability of 25.6% was the difference between the two HCC incidences (41.1% and 15.5%).

The LRT between the original model and model revision (p-value < 0.001) highlighted the need for recalibration [15]. Recalibration-in-the-large yielded comparable performance to model revision (LRT χ2 p-value = 0.332), e.g., the same excellent discrimination, good calibration, and increased NB across a wide range of probability thresholds (Fig. 2b, c), avoiding the excessive and unnecessary measurements that would be undertaken if the original model was used. Additionally, the updated model had superior accuracy and clinical usefulness in patients at risk for HCC with single HBV infections than in those with other etiologies.

The hierarchy of the quality of the model external verification using new data from an independent research institution is better than temporal verification and geographic verification using data collected by the original research team for model development. Model verification with new data might lead to decreased accuracy and miscalibration and might influence the model outcome. However, we could adjust the overfitting, improve the model's calibration and overall prediction performance, and use the model in diverse populations and settings [22, 23]. Our data were collected from a different population and clinical scenario from that of the development cohort. We corrected the calibration by adding an intercept of − 3.243 to the original ASAP model, thus promoting its generalizability. Our research provided a robust verification of the ASAP model.

Accurate predictions and precise interventions have received increasing attention. Personalization surveillance schemes are expected to benefit patients by facilitating the early detection of HCC, while risk-adapted interventions are expected to prevent or delay the progression of HCC and improve patient prognosis [4, 24, 25]. The absence of valid noninvasive risk stratification tools makes the personalized management of at-risk patients challenging. The risk thresholds in a prediction model are commonly determined arbitrarily [10] and seldom further tested with regard to their impact on clinical outcomes [7, 26]. Proper thresholds can benefit clinical management strategies, while inappropriate thresholds negatively affect decision-making processes [10]. The thresholds set for the online ASAP nomogram, 1/3 and 2/3, were somewhat unsuitable for the recalibration-in-the-large to risk stratification in clinical contexts similar to ours, which led to poor predictive results at the follow-up outcome evaluation, i.e., most patients were in the low-risk group, there were fewer medium-risk patients than high-risk patients (32 vs. 124), and there was a higher cumulative HCC incidence rate in the low-risk group than in the medium-risk group (see Additional file 6: Fig. S4). The choice of risk thresholds, discrimination, calibration, and clinical utility need to be reevaluated before its application in a new clinical setting. We assessed the performance of recalibration-in-the-large at multiple probability thresholds (Table 3) [10, 26] and chose a relatively stable median value of non-HCC cases and a less harmful event rate to group patients according to their level of risk [11, 18] with reference to the distribution characteristics of the predictive values in the study cohort (see Additional file 4: Fig. S3b). The probability thresholds of 1.3% and 15.5% reasonably and effectively defined the risk categories (Table 3 and Fig. 3). Thus, the prediction model laid a foundation for the further clinical application of risk stratification to better manage targeted individuals [26, 27].

Routine semiannual follow-up monitoring in at-risk patients was expected to improve the detection of early-stage HCC and the clinical outcomes [3, 20, 28, 29]. Low-risk populations with an annual incidence rate of HCC < 0.2% can be exempted from surveillance [28, 30, 31]. However, current studies have shown that the clinical effects are unclear for various reasons [3, 20]. First, the limited compliance of patients was not satisfactory [4]. Second, the one-size-fits-all recommendation was unlikely to be applicable to all patients in multiple settings [32]. Recently, Rich NE et al reported that tumor growth has obvious heterogeneity, and the tumor doubling time varies from less than 3 months to more than half a year [33]. 3-month diagnostic delays can allow tumors to grow significantly, leading to less effective treatment [34]. The expert consensus in East Asia also pointed out that HBV-infected patients with two risk factors for HCC development need to initiate antiviral therapy or undergo monitoring at 3-month intervals [25]. Third, the 6-month or unclear detection time window in most HCC risk prediction models leads to decreased predictive performance of models based on tumor markers that fluctuate over time [14] and ineffective suggestions regarding the need for 3-month follow-up visits. Our follow-up outcome analysis showed that among the low-risk group, which had an HCC diagnosis rate of 0%, at least 0.6% of the individuals should return at 3 months to facilitate the discrimination of very early-stage HCC from atypical hyperplastic nodules. Three-month follow-up strategies developed for the small proportion of the population with nodules of uncertain identity will facilitate the early diagnosis of HCC. The evaluation of the clinical impact of predictive models helps refine the follow-up strategies for different risk groups.

Routine serum biomarkers are often helpful in assessing the HCC risk of patients. Despite its limited accuracy, AFP is the most commonly used HCC diagnostic biomarker due to its increase in the early stages of the disease. PIVKA-II is another widely used HCC marker with high specificity. PIVKA-II is a powerful supplement to AFP in HCC diagnosis [35], and their combination could improve the accuracy [35–38]. Recent reports have suggested that the ratio of gamma-glutamyltransferase to aspartate aminotransferase (γ-GT/AST) and the *Lens culinaris* agglutinin-reactive fraction of AFP (AFP-L3) are valuable markers in HCC diagnosis. The addition of the γ-GT/AST ratio or AFP-L3 to the combination of AFP and PIVKA-II could further improve the accuracy of HCC diagnosis [36, 37]. Our data show that the γ-GT/AST ratio of the patients with HCC was significantly higher than that of the other risk groups (p-value < 0.05) (Table 1). However, when combined with AFP and PIVKA-II, the γ-GT/AST ratio did not significantly contribute to the logistic regression models predicting HCC (p-value = 0.835) or early HCC (p-value = 0.716). AFP-L3 has been reported to be a sensitive indicator for early HCC detection, and its diagnostic performance was inferior to AFP and PIVKA-II [37, 38]. Considering its cost-effectiveness and relatively lower performance in HCC prediction, few studies have reported the effectiveness of AFP-L3 combined with AFP and PIVKA-II in the diagnosis of overall and early HCC [8, 37]. In the existing reports, the increment value of the C-statistic of AFP-L3 to the model of AFP and PIVKA-II remains controversial [37, 38].

Our analysis showed that HCC occurrence was most closely related to hepatitis B virus infection; the incidences of HCC in populations with different etiologies were similar (p-value > 0.05). The simpler ASAP model that only included the AFP and PIVKA-II levels and two patient observable attributes, such as age and sex [8], did not need to detect other indexes, was not limited by the etiologies of liver disease and the antiviral status, could accurately predict the risk of HCC and was easily validated and calibrated with new data from diverse settings.

Our research is the first to apply the HCC diagnostic model as a prognostic management tool to the population at risk of HCC. This model efficiently excavated the potential predictive function of the ASAP diagnostic model. This model helped make clinical follow-up decisions for different risk populations, which could achieve long-term and refined management of patients at risk for HCC. Based on the risk thresholds selected based on the population distribution characteristics, high-risk groups could receive more attention and monitoring, and the allocation of medical resources could be optimized, which could be beneficial for the early detection, intervention, diagnosis, and treatment of HCC. This model could improve the prognosis of at-risk patients for HCC. Moreover, these indicators have the characteristics of noninvasiveness, easy availability, repeatability, and standardization, which are conducive to applying and promoting the model in clinical practice.

### Strength

We determined three weeks as the test-to-diagnosis interval based on the clinical characteristics of HCC diagnosis [12, 13], which increased the performance of the model and provided evidence supporting the development of 3-month follow-up strategies and patient counseling. We improved the applicability of the ASAP model to patients at risk for HCC regardless of etiology by updating it based on data from a real-world cohort. We clarified that stratified management with reasonable thresholds had the potential to aid in achieving an early diagnosis and avoiding clinical harm. We refined the personalized recommendations for different risk groups through a follow-up analysis, e.g., definitively low-risk patients can prolong their follow-up intervals to one year, avoiding unnecessary investigations, enabling the allocation of limited medical resources to the most high-risk patients, which is an especially important consideration during the coronavirus disease 2019 pandemic.

### Limitations

A total of 18.8% (161/855) of the patients were lost to follow-up, which might have resulted in inaccurate estimates concerning the associations between HCC occurrence and the predicted risk. The current study focused on the prediction of the risk of all-cause HCC; however, the risk of HCC differs by etiology [3, 4, 28, 29]. It would be better to generate recalibrated models for each specific etiology, which would necessitate a larger sample size. The models appear to be unable to identify the risk of very early-stage cases due to their relatively low proportion in the study and the large overlap in the AFP and PIVKA-II levels between early-stage HCC and the risk groups (Additional file 2: Fig. S1a, b). Nevertheless, the addition of an appropriate risk classification strategy to the high discrimination ability of the predictive model could effectively improve the status quo. The classification of patients with indeterminate nodules into the high-risk group, thereby ensuring that these patients undergo intensive surveillance, may address this shortcoming [4]. Validation was performed at a single center with limited data, and no cost-benefit analysis was performed.

## Conclusion

The ASAP model can be used to accurately predict incident HCC and facilitate the personalized management of at-risk patients. However, independent and robust model validation is essential to ensure that the appropriate tools are available for the precise prediction of incident HCC, which can be used during patient consultation and intervention decision-making in novel clinical settings. The well-calibrated model obtained with recalibration-in-the-large combined with the risk-stratified strategies established in the article is suitable for the noninvasive monitoring of patients at risk for HCC in primary care centers. The overall performance needs further validation and refinement in larger cohorts. Developing region-specific recalibration models and selecting the corresponding risk thresholds may improve the generalizability of the model.

## Supplementary Information

**Additional file 1:Table S1.** TRIPOD checklist: Prediction model validation.

**Additional file 2:Fig. S1.** Serum levels of AFP and PIVKA-II. Serum levels of AFP (**a**) and PIVKA-II (**b**) in the five subgroups of the validation cohort. In each plot, each subgroup was compared with the median level of the whole cohort. Differences between subgroups are shown (ns: not significant; *: p-value < 0.05; **: p-value < 0.01; ***: p-value < 0.001). Abbreviations: AFP, alpha-fetoprotein; HCC, hepatocellular carcinoma; PIVKA-II, prothrombin induced by vitamin K absence-II; SOL, space-occupying lesion.

**Additional file 3: Fig. S2.** Calibration plots of the predicted probabilities versus the observed probabilities of (**a**) recalibration and (**b**) model revision derived using the method described by Vergouwe et al (15) in the validation cohort (n = 1012). A nonparametric calibration curve with its 95% CL (red slide line with dashed lines) was created with the Loess algorithm. Observed HCC occurrence (green triangles) with the 95% CL was plotted against the average predicted probability in each group. The blue straight diagonal line serves as a reference for perfect calibration. The brown bar chart at the bottom of the figure presents the distribution of the predicted probabilities of the cases with outcomes (above the line) and those without outcomes (below the line) (“1” vs. “0”). Abbreviations: CL: confidence limits; HCC: hepatocellular carcinoma; Loess: locally weighted linear regression.

**Additional file 4:Fig. S3.** Nomogram of the model revision and risk probability threshold selection. Description of data: **a** Nomogram of the model revision predicting HCC risk. Although sex was not significant in the revised model, we retained this variable in the revision nomogram due to its clinical relevance and high relative frequency in most models. The logit (P) calculation formula is {-12.95521 + 0.06178[age] - 0.7449[sex]+ 0.55643[log(AFP)] + 1.28268[log(PIVKA-II)]}. The prediction probability (P) calculation formula is exp[logit (P)]/{1+ exp[logit (P)]}. The illustrated patient #39 maps its values to the covariate scales. The calculated nomogram score was 95.1 points, and the estimated prediction probability of HCC was 0.177 (95% CI, 0.102-0.288). Tables of point assignments by levels of predictors are shown in Additional file 5: Table S2. **b** Predicted probability of HCC versus the densities of the non-HCC and HCC patients in the validation cohort. Based on the relationship between the prediction probability and nomogram score of the patients with non-HCC and HCC, the patients were divided into low-, medium-, high-, and very high- risk groups by the following three thresholds: the median of non-HCC patients due to its relative stability, the value of the incidence rate with less clinical harm (11), and the median of HCC cases capable of identifying very high-risk patients. Their corresponding cutoff scores were 72.5 points (the median of non-HCC patients), 94 points (the incidence in the cohort), and 138 points (the median of HCC patients). Abbreviations: ***: p < 0.001; AFP: α-fetoprotein; CI: confidence interval; HCC: hepatocellular carcinoma; PIVKA-II: protein induced by vitamin K absence or antagonist-II; Pr/Prob.: probability.

**Additional file 5:Table S2. **Nomogram score tables of the model revision.

**Additional file 6:Fig. S4.** Cumulative HCC incidences in the recalibration-in-the-large-estimated risk groups classified by the tertile thresholds. Kaplan-Meier curve demonstrating significant differences in the cumulative HCC incidences among the low-risk group (predicted probability < 1/3), medium-risk group (predicted probability 1/3–2/3), and high-risk group (predicted probability > 2/3) (log-rank test, p-value = 0.016) based on the estimation of recalibration-in-the-large. The cumulative incidence in the medium-risk group was the lowest and lower than that in the low-risk group. The two probability thresholds were suboptimal for risk classification based on the estimation by recalibration-in-the-large in the validation cohort. Abbreviations: HCC, hepatocellular carcinoma.

## Data Availability

The datasets used and analyzed in the current study are available from the corresponding author upon reasonable request.

## Abbreviations

AFP: alpha-fetoprotein
AFP-L3: the *Lens culinaris* agglutinin-reactive fraction of AFP
AIC: akaike information criterion
BCLC: Barcelona Clinic Liver Cancer
CI: confidence interval
CL: confidence limits
df: degrees of freedom
Eavg: average absolute difference in the predicted and calibrated probabilities
Emax: maximum absolute difference in the predicted and calibrated probabilities
γ-GT/AST: the ratio of gamma-glutamyltransferase to aspartate aminotransferase
HBeAg: hepatitis B virus e antigen
HBV: hepatitis B virus
HC: healthy control
HCC: hepatocellular carcinoma
HCV: hepatitis C virus
HEV: hepatitis E virus
Loess: locally weighted linear regression
LRT: likelihood ratio test
NB: net benefit
NPV: negative predictive value
PIVKA-II: prothrombin induced by vitamin K absence-II
PPV: positive predictive value
Pr/Prob.: probability
RR: relative risk
SD: standard deviation
SOL: space-occupying lesion

## Code availability

The custom code used to analyze the data are available from the corresponding author upon reasonable request.
