# Supplementary figures and images for: Validation and update of a multivariable prediction model for the identification and management of patients at risk for hepatocellular carcinoma
Source: Clin Proteomics. 2021 Aug 19;18:21. doi: 10.1186/s12014-021-09326-w (PMC8374120; doi:10.1186/s12014-021-09326-w)

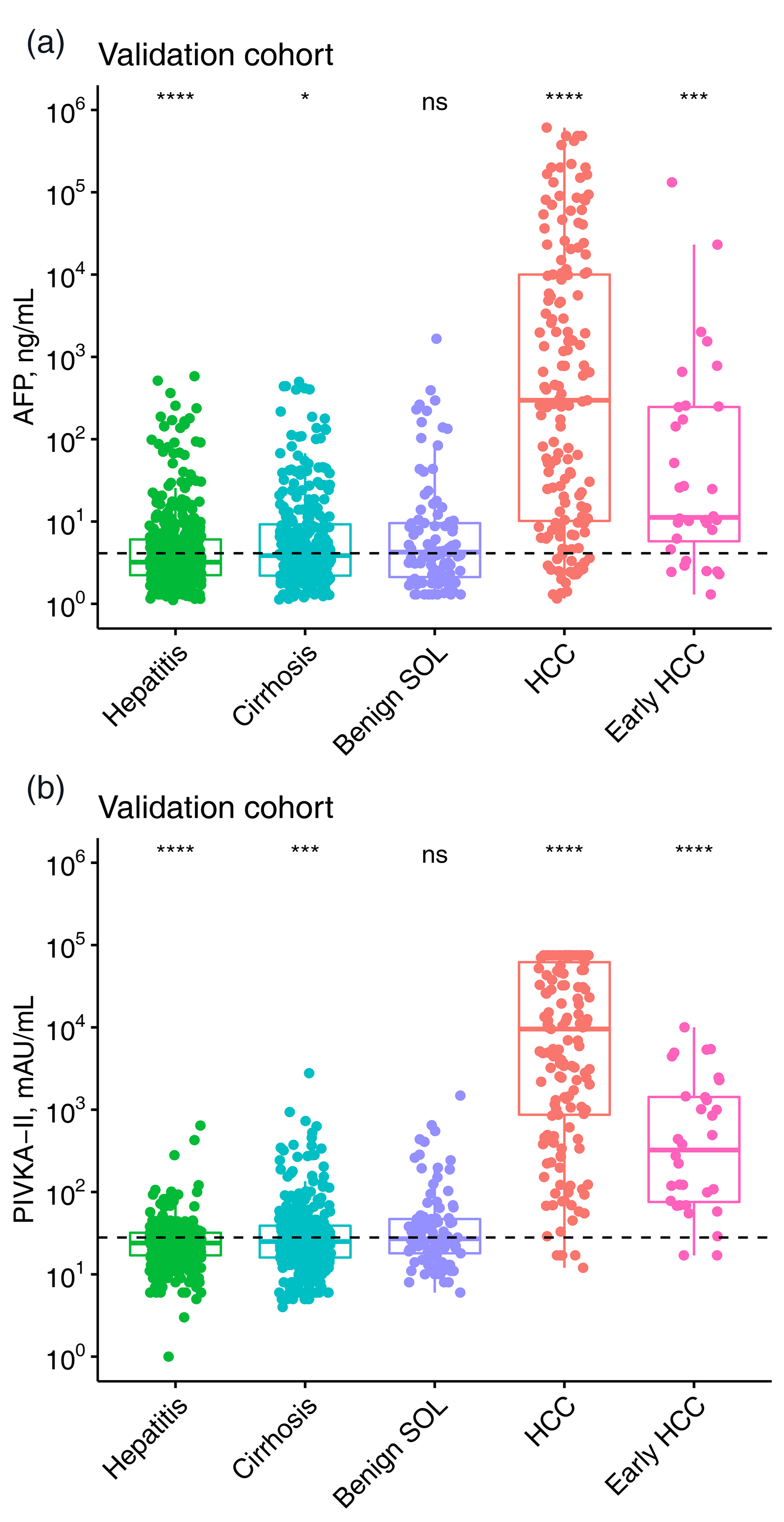

Supplement: Supplementary file 2 — Additional file 2:Fig. S1. Serum levels of AFP and PIVKA-II. Serum levels of AFP (a) and PIVKA-II (b) in the five subgroups of the validation cohort. In each plot, each subgroup was compared with the median level of the whole cohort. Differences between subgroups are shown (ns: not significant; *: p-value < 0.05; **: p-value < 0.01; ***: p-value < 0.001). Abbreviations: AFP, alpha-fetoprotein; HCC, hepatocellular carcinoma; PIVKA-II, prothrombin induced by vitamin K absence-II; SOL, space-occupying lesion. [file 12014_2021_9326_MOESM2_ESM.tiff]

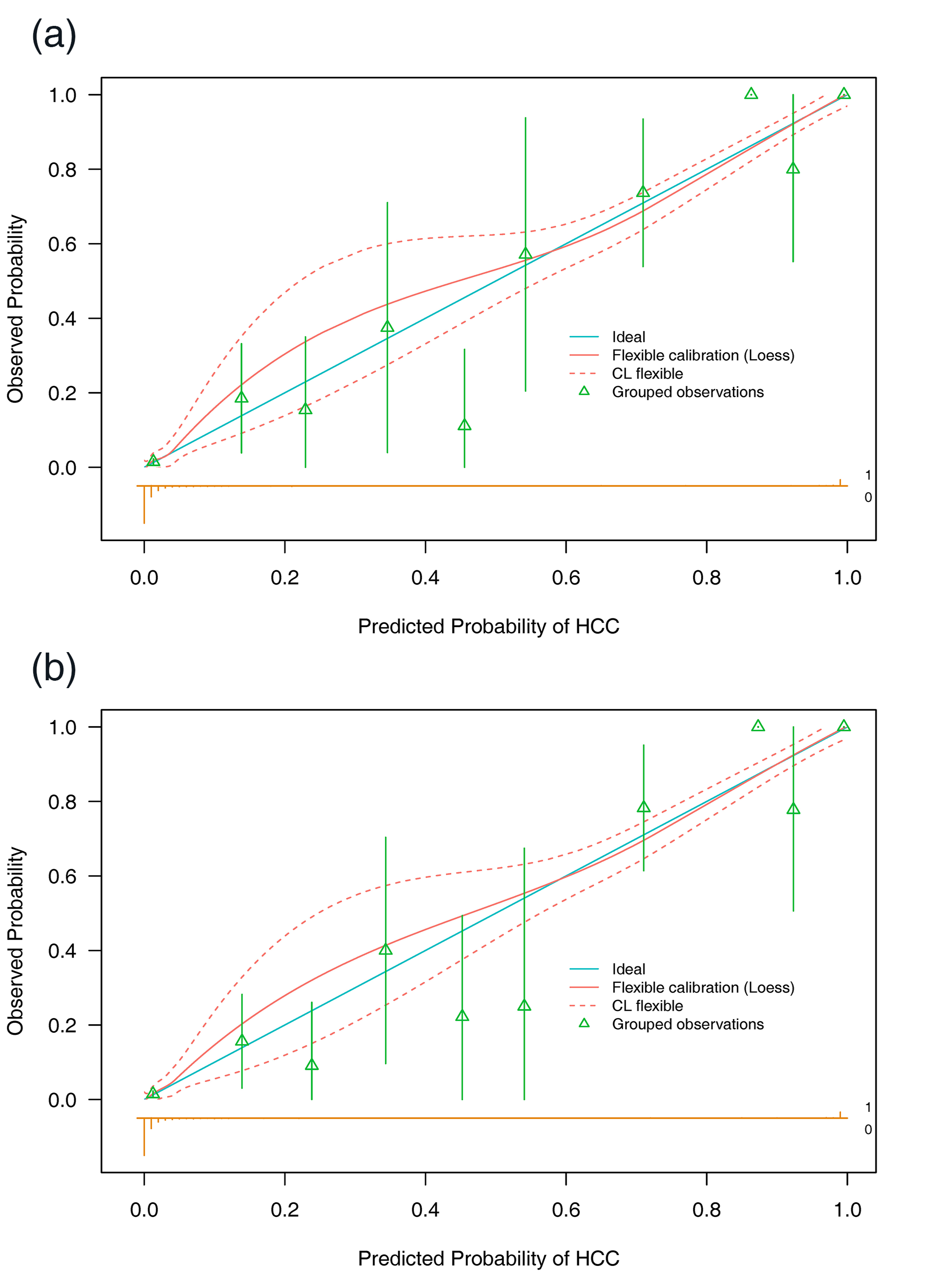

Supplement: Supplementary file 3 — Additional file 3: Fig. S2. Calibration plots of the predicted probabilities versus the observed probabilities of (a) recalibration and (b) model revision derived using the method described by Vergouwe et al (15) in the validation cohort (n = 1012). A nonparametric calibration curve with its 95% CL (red slide line with dashed lines) was created with the Loess algorithm. Observed HCC occurrence (green triangles) with the 95% CL was plotted against the average predicted probability in each group. The blue straight diagonal line serves as a reference for perfect calibration. The brown bar chart at the bottom of the figure presents the distribution of the predicted probabilities of the cases with outcomes (above the line) and those without outcomes (below the line) (“1” vs. “0”). Abbreviations: CL: confidence limits; HCC: hepatocellular carcinoma; Loess: locally weighted linear regression. [file 12014_2021_9326_MOESM3_ESM.tiff]

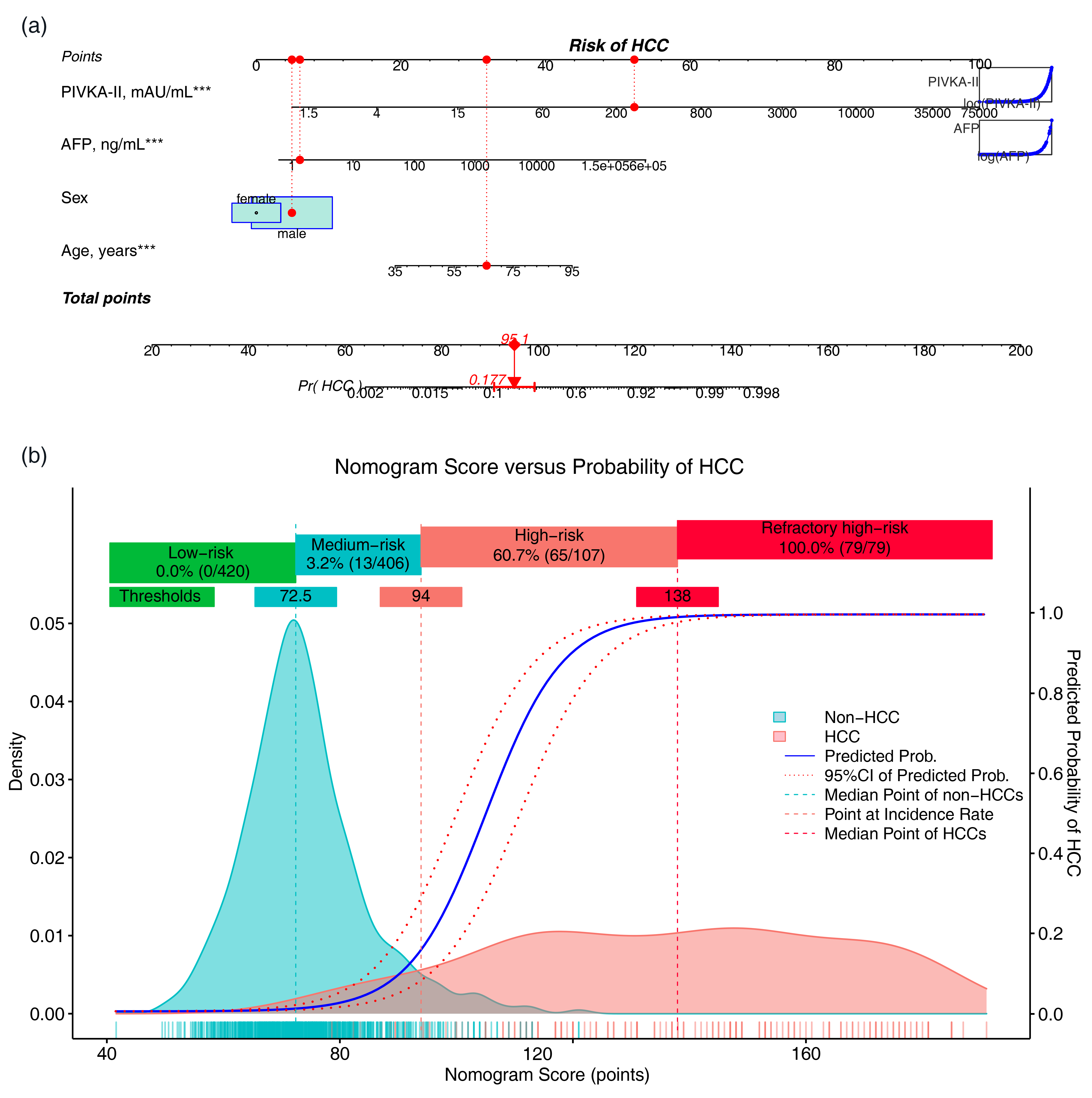

Supplement: Supplementary file 4 — Additional file 4:Fig. S3. Nomogram of the model revision and risk probability threshold selection. Description of data: a Nomogram of the model revision predicting HCC risk. Although sex was not significant in the revised model, we retained this variable in the revision nomogram due to its clinical relevance and high relative frequency in most models. The logit (P) calculation formula is {-12.95521 + 0.06178[age] - 0.7449[sex]+ 0.55643[log(AFP)] + 1.28268[log(PIVKA-II)]}. The prediction probability (P) calculation formula is exp[logit (P)]/{1+ exp[logit (P)]}. The illustrated patient #39 maps its values to the covariate scales. The calculated nomogram score was 95.1 points, and the estimated prediction probability of HCC was 0.177 (95% CI, 0.102-0.288). Tables of point assignments by levels of predictors are shown in Additional file 5: Table S2. b Predicted probability of HCC versus the densities of the non-HCC and HCC patients in the validation cohort. Based on the relationship between the prediction probability and nomogram score of the patients with non-HCC and HCC, the patients were divided into low-, medium-, high-, and very high- risk groups by the following three thresholds: the median of non-HCC patients due to its relative stability, the value of the incidence rate with less clinical harm (11), and the median of HCC cases capable of identifying very high-risk patients. Their corresponding cutoff scores were 72.5 points (the median of non-HCC patients), 94 points (the incidence in the cohort), and 138 points (the median of HCC patients). Abbreviations: ***: p < 0.001; AFP: α-fetoprotein; CI: confidence interval; HCC: hepatocellular carcinoma; PIVKA-II: protein induced by vitamin K absence or antagonist-II; Pr/Prob.: probability. [file 12014_2021_9326_MOESM4_ESM.tiff]

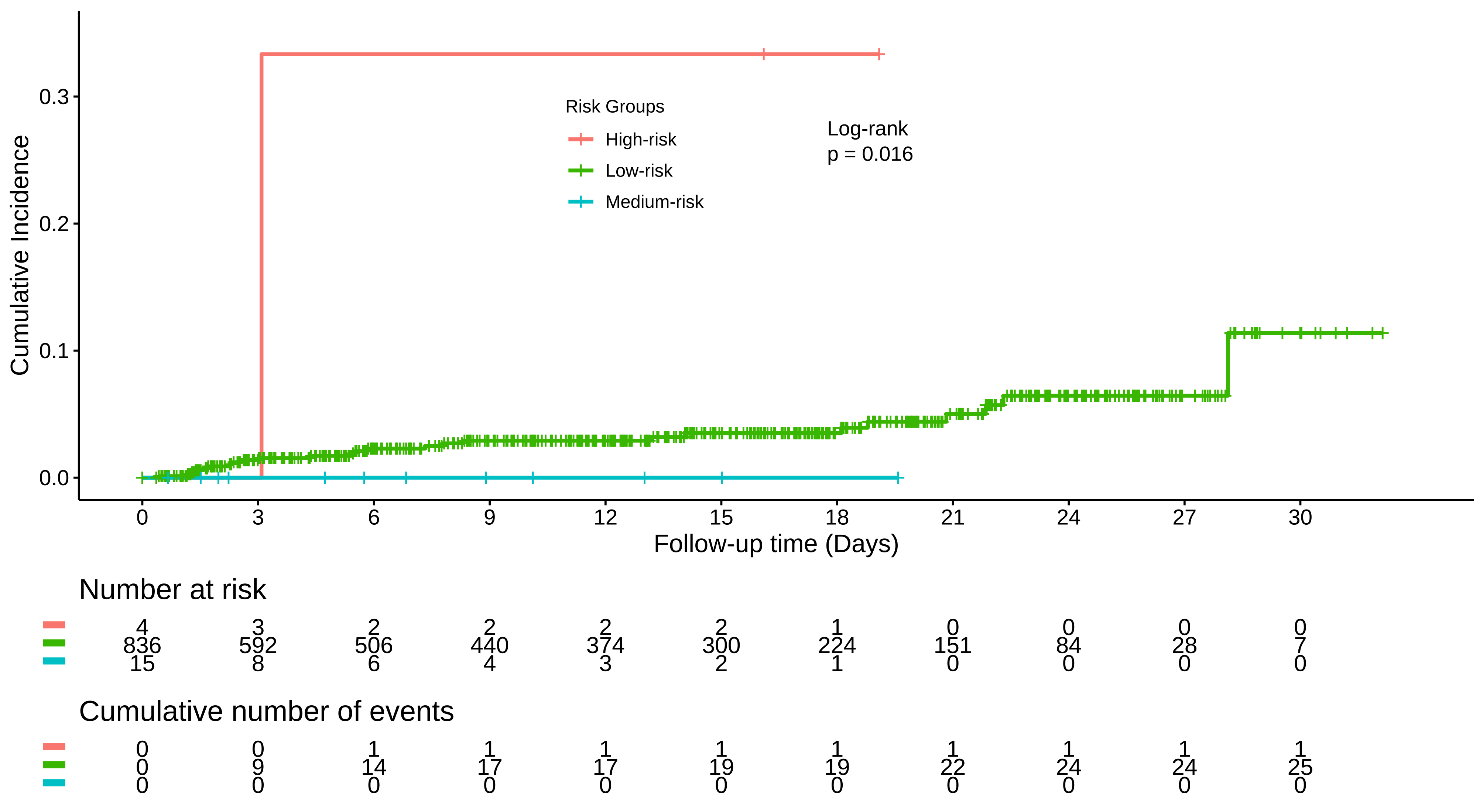

Supplement: Supplementary file 6 — Additional file 6:Fig. S4. Cumulative HCC incidences in the recalibration-in-the-large-estimated risk groups classified by the tertile thresholds. Kaplan-Meier curve demonstrating significant differences in the cumulative HCC incidences among the low-risk group (predicted probability < 1/3), medium-risk group (predicted probability 1/3–2/3), and high-risk group (predicted probability > 2/3) (log-rank test, p-value = 0.016) based on the estimation of recalibration-in-the-large. The cumulative incidence in the medium-risk group was the lowest and lower than that in the low-risk group. The two probability thresholds were suboptimal for risk classification based on the estimation by recalibration-in-the-large in the validation cohort. Abbreviations: HCC, hepatocellular carcinoma. [file 12014_2021_9326_MOESM6_ESM.tiff]
