# Supplementary material for: Validation and update of a multivariable prediction model for the identification and management of patients at risk for hepatocellular carcinoma
Source: Clin Proteomics. 2021 Aug 19;18:21. doi: 10.1186/s12014-021-09326-w (PMC8374120; doi:10.1186/s12014-021-09326-w)
Supplement: Supplementary file 5 — Additional file 5:Table S2. Nomogram score tables of the model revision. [file 12014_2021_9326_MOESM5_ESM.docx]

**Supplement Table 2** Nomogram score tables of model revision

[[1]]

**PIVKA_II (mAU/mL) Points**

1 1.5 7

2 4.0 17

3 15.0 28

4 60.0 40

5 200.0 50

6 800.0 62

7 3000.0 73

8 10000.0 83

9 35000.0 94

10 75000.0 100

[[2]]

**AFP (ng/mL) Points**

1 1 5

2 10 13

3 100 22

4 1000 30

5 10000 39

6 150000 49

7 600000 54

[[3]]

**Sex Points**

Sex2 female 0

Sex1 male 5

[[4]]

**Age (years) Points**

1 35 19

2 55 27

3 75 36

4 95 44

[[5]]

**Total Points Pr( HCC )**

1 0 1.201e-07

2 20 2.483e-06

3 40 5.131e-05

4 60 1.060e-03

5 80 2.145e-02

6 100 3.118e-01

7 120 9.035e-01

8 140 9.949e-01

9 160 9.998e-01

10 180 1.000e+00

11 200 1.000e+00

12 220 1.000e+00
